# Supplementary material for: A Foxf1-Wnt-Nr2f1 cascade promotes atrial cardiomyocyte differentiation in zebrafish
Source: bioRxiv. 2024 Mar 19:2024.03.13.584759. Preprint. [Version 2] doi: 10.1101/2024.03.13.584759 (PMC10980076; doi:10.1101/2024.03.13.584759)
Supplement: Supplement 2 [file media-2.docx]

**S1 Table. *Danio rerio* promoter sequences used in transgenic constructs. cloned in GFP vector with their names and genomic coordinates, respectively.**

>-1.5_nr2f1a_5:49743133-49744948

TTGTGTTCCCATTCGCTGTCCCTCTCCCTCTCTCCCCTCTCGCTCTCTTCCCTATCTGTGGAGTAAAACCTTGATGGTACACTTACAGAAGTTTCTCTTTGTTCGTTTTAATCTTATTCGTTTCACCTTTGCAATGAACAGAGGACAACAGCGTGTGTTTCTATGGATCTGTTTGTGGAGGGACGGAGAAGCTCCGCGGTCGAGTTGAAAATAAGCTACCTTTTTGTAACTAGAGGGTTTCTTGAACGGTCGGGTATCGGGGTTTTGTTTCCTCTTAACTTCACTTACACAGTTTTGGACTTTGATGCATTGTTTGTACAAAAAAAAAAAAAAACTTATGGTAAAAATAGGTGTTAGGTGATAAAGGCTACTAAAATATTTATTAAATACTTGAATTTTAAACATTGTTGTTTGTCCGCTAATAAGAGGCCAAAAAGCTTTTCTCCGAAAACAAAAATAAAATAAGCATCTCAAGTGGAGACATTGATAAAAAATAAAAATAAACTTGATGCTAAACAAGTTCCTGCAAAACATAATCAAAATACTTTTAAATCTGATGTTTTTAATAGCCTACTTCACTAAAAAAAATCAGAAACATTCATCCCACCGCGCAATTTGTCTACGAGTTACATTTGGAAACATGCTTTAAATGACAATAAATTACTAATTTTTCATGTCGCTGTAATTGTCTACTGTACGCTAGATGGCAGCCCGATCCAGACATTATAAGTCTGTACAGCGCAGCCTTCAGCATTCATGCTCAAAAAGCCGGTTTCCCTGCGCATTATACCAAAACTTCCCTCGTTAATAAATATGTTAAAACATGGATGGATCCATCGATGTATTTTTCTATTTTTCAATATCATTATACTTTGATTCCTCTTTTGTGCCCTGGTTTACTGCTTGATATGTTTCCTATCACTGAGAAAACATTCCGTTATAGCCTATACTGATATATCCCTCTAGTCAAACTAGTGGTAGTCATCGCGCTTCCCGGTGCTTTTTAATTTAATCGTTGTGCTGAGGATTAAGTGTAATATTGACACGTAAAAGCGAATTTTATACATCGATACGCAATTTCGAGTCGGTGGGCTATATCGGTCTGTCAAATTGCGTTAAACATTAATGGGACCTTCATAGTTCAAATCTGGACGCCGTTACGGTGTTTTTGTGTAATTAAGACTAGGGCTTTGAACAAAACACTTTCAACTTTGTGAGGTTCGCACCAGTCCTTTTGCCTCACACGCATTTGTTTATTTTTCACGTGCGCGTAGTTCGCTCATTGCTTTCATCCCGCTTTTCGCCGCTTCTTTCTGGGGTTTCTTCCTCTTTTTTTGTGACAATAGACGGTCCATTCTAATCGGGTTTCAAAGTAAATAGCAGCGCAGGGGCGAGAGCAATGTAAATTGTGCTTGTCAGAGCCCTCAGTCGTAGGTAGCGAGGAGCGAGGACTCTAACCAATGGAGTGAAGGAGGCTTGGCTAACCTTAGCCTCCCATTTTCTCTCTCCCCCCTCGATGCAGGGTTCTGACCAGTCAGTCGCCTTTGATTATCAGTTGCCAGCAGCCCCTCTCTTGGCTCCTTGACACGAGCACCATATAAGGCGCAGCGATCTCCATAGAAACGTGTCAGTTTCAATAGTAGTGTCAAAGTTCACTATATACAGACATTCGCGCAGATCTCCGTTTCGGAAACATTGCTCCGCTGGTACTCCCGTTAAAACGCATTCTTTTTGGGTCTCTGCTTCTTACATATTCCATTAGTTGCTTTTTTTCTTTTCTTTTCCTACTGGAGAGGTGAAACTACATAGCC

>-1.4_nr2f1a_5:49743263-49744948

GCAATGAACAGAGGACAACAGCGTGTGTTTCTATGGATCTGTTTGTGGAGGGACGGAGAAGCTCCGCGGTCGAGTTGAAAATAAGCTACCTTTTTGTAACTAGAGGGTTTCTTGAACGGTCGGGTATCGGGGTTTTGTTTCCTCTTAACTTCACTTACACAGTTTTGGACTTTGATGCATTGTTTGTACAAAAAAAAAAAAAACTTATGGTAAAAATAGGTGTTAGGTGATAAAGGCTACTAAAATATTTATTAAATACTTGAATTTTAAACATTGTTGTTTGTCCGCTAATAAGAGGCCAAAAAGCTTTTCTCCGAAAACAAAAATAAAATAAGCATCTCAAGTGGAGACATTGATAAAAAATAAAAATAAACTTGATGCTAAACAAGTTCCTGCAAAACATAATCAAAATACTTTTAAATCTGATGTTTTTATTAGCCTACTTCACTAAAAAAAATCAGAAACATTCATCCCACCGCGCAATTTGTCTACGAGTTACATTTGGAAACATGCTTTAAATGACAATAAATTACTAATTTTTCATGTCGCTGTAATTGTCTACTGTACGCTAGATGGCAGCCCGATCCAGACATTATAAGTCTGTACAGCGCAGCCTTCAGCATTCATGCTCAAAAAGCCGGTTTCCCTGCGCATTATACCAAAACTTCCCTCGTTAATAAATATGTTAAAACATGGATGGATCCATCGATGTATTTTTCTATTTTTCAATATCATTATACTTTGATTCCTCTTTTGTGCCCTGGTTTACTGCTTGATATGTTTCCTATCACTGAGAAAACATTCCGTTATAGCCTATACTGATATATCCCTCTAGTCAAACTAGTGGTAGTCATCGCGCTTCCCGGTGCTTTTTAATTTAATCGTTGTGCTGAGGATTAAGTGTAATATTGACACGTAAAAGCGAATTTTATACATCGATACGCAATTTCGAGTCGGTGGGCTATATCGGTCTGTCAAATTGCGTTAAACATTAATGGGACCTTCATAGTTCAAATCTGGACGCCGTTACGGTGTTTTTTTGTAATTAAGACTAGGGCTTTGAACAAAACACTTTCAACTTTGTGAGGTTCGCACCAGTCCTTTTGCCTCACACGCATTTGTTTATTTTTCACGTGCGCGTAGTTCGCTCATTGCTTTCATCCCGCTTTTCGCCGCTTCTTTCTGGGGTTTCTTCCTCTTTTTTTGTGACAATAGACGGTCCATTCTAATCGGGTTTCAAAGTAAATAGCAGCGCAGGGGCGAGAGCAATGTAAATTGTGCTTGTCAGAGCCCTCAGTCGTAGGTAGCGAGGAGCGAGGACTCTAACCAATGGAGTGAAGGAGGCTTGGCTAACCTTAGCCTCCCATTTTCTCTCTCCCCCCTCGATGCAGGGTTCTGACCAGTCAGTCGCCTTTGATTATCAGTTGCCAGCAGCCCCTCTCTTGGCTCCTTGACACGAGCACCATATAAGGCGCAGCGATCTCCATAGAAACGTGTCAGTTTCAATAGTAGTGTCAAAGTTCACTATATACAGACATTCGCGCAGATCTCCGTTTCGGAAACATTGCTCCGCTGGTACTCCCGTTAAAACGCATTCTTTTTGGGTCTCTGCTTCTTACATATTCCATTAGTTGCTTTTTTCTTTTCTTTTCCTACTGGAGAGGTGAAACTACATAGCC

>-0.7s_nr2f1a_5:49744016-49744948

CCTCTTTTGTGCCCTGGTTTACTGCTTGATATGTTTCCTATCACTGAGAAAACATTCCGTTATAGCCTATACTGATATATCCCTCTAGTCAAACTAGTGGTAGTCATCGCGCTTCCCGGTGCTTTTTAATTTAATCGTTGTGCTGAGGATTAAGTGTAATATTGACACGTAAAAGCGAATTTTATACATCGATACGCAATTTCGAGTCGGTGGGCTATATCGGTCTGTCAAATTGCGTTAAACATTAATGGGACCTTCATAGTTCAAATCTGGACGCCGTTACGGTGTTTTTGTGTAATTAAGACTAGGGCTTTGAACAAAACACTTTCAACTTTGTGAGGTTCGCACCAGTCCTTTTGCCTCACACGCATTTGTTTATTTTTCACGTGCGCGTAGTTCGCTCATTGCTTTCATCCCGCTTTTCGCCGCTTCTTTCTGGGGTTTCTTCCTCTTTTTTTGTGACAATAGACGGTCCATTCTAATCGGGTTTCAAAGTAAATAGCAGCGCAGGGGCGAGAGCAATGTAAATTGTGCTTGTCAGAGCCCTCAGTCGTAGGTAGCGAGGAGCGAGGACTCTAACCAATGGAGTGAAGGAGGCTTGGCTAACCTTAGCCTCCCATTTTCTCTCTCCCCCCTCGATGCAGGGTTCTGACCAGTCAGTCGCCTTTGATTATCAGTTGCCAGCAGCCCCTCTCTTGGCTCCTTGACACGAGCACCATATAAGGCGCAGCGATCTCCATAGAAACGTGTCAGTTTCAATAGTAGTGTCAAAGTTCACTATATACAGACATTCGCGCAGATCTCCGTTTCGGAAACATTGCTCCGCTGGTACTCCCGTTAAAACGCATTCTTTTTTGGGTCTCTGCTTCTTACATATTCCATTAGTTGCTTTTTTTCTTTTCTTTTCCTACTGGAGAGGTGAAACTACATAGCC

>-0.7m_nr2f1a_5:49744016-49745075

CCTCTTTTGTGCCCTGGTTTACTGCTTGATATGTTTCCTATCACTGAGAAAACATTCCGTTATAGCCTATACTGATATATCCCTCTAGTCAAACTAGTGGTAGTCATCGCGCTTCCCGGTGCTTTTTAATTTAATCGTTGTGCTGAGGATTAAGTGTAATATTGACACGTAAAAGCGAATTTTATACATCGATACGCAATTTCGAGTCGGTGGGCTATATCGGTCTGTCAAATTGCGTTAAACATTAATGGGACCTTCATAGTTCAAATCTGGACGCCGTTACGGTGTTTTTTTGTAATTAAGACTAGGGCTTTGAACAAAACACTTTCAACTTTGTGAGGTTCGCACCAGTCCTTTTGCCTCACACGCATTTGTTTATTTTTCACGTGCGCGTAGTTCGCTCATTGCTTTCATCCCGCTTTTCGCCGCTTCTTTCTGGGGTTTCTTCCTCTTTTTTTGTGACAATAGACGGTCCATTCTAATCGGGTTTCAAAGTAAATAGCAGCGCAGGGGCGAGAGCAATGTAAATTGTGCTTGTCAGAGCCCTCAGTCGTAGGTAGCGAGGAGCGAGGACTCTAACCAATGGAGTGAAGGAGGCTTGGCTAACCTTAGCCTCCCATTTTCTCTCTCCCCCCTCGATGCAGGGTTCTGACCAGTCAGTCGCCTTTGATTATCAGTTGCCAGCAGCCCCTCTCTTGGCTCCTTGACACGAGCACCATATAAGGCGCAGCGATCTCCATAGAAACGTGTCAGTTTCAATAGTAGTGTCAAAGTTCACTATATACAGACATTCGCGCAGATCTCCGTTTCGGAAACATTGCTCCGCTGGTACTCCCGTTAAAACGCATTCTTTTTTGGGTCTCTGCTTCTTACATATTCCATTAGTTGCTTTTTTTCTTTTCTTTTCCTACTGGAGAGGTGAAACTACATAGCCAGTCGGGCGACGTTGCTTTTTTTCGCTGGACCAGATGAGCTTTATTCATGAACATAGATAGAGAAATCCGTTCTTCAGTGTTTCCTCCTCTCCAACCGCGAAGACGGAGAGAGGAGCAAGGAAGAAA

>-0.7l_nr2f1a_5:49744016-49745426

CCTCTTTTGTGCCCTGGTTTACTGCTTGATATGTTTCCTATCACTGAGAAAACATTCCGTTATAGCCTATACTGATATATCCCTCTAGTCAAACTAGTGGTAGTCATCGCGCTTCCCGGTGCTTTTTAATTTAATCGTTGTGCTGAGGATTAAGTGTAATATTGACACGTAAAAGCGAATTTTATACATCGATACGCAATTTCGAGTCGGTGGGCTATATCGGTCTGTCAAATTGCGTTAAACATTAATGGGACCTTCATAGTTCAAATCTGGACGCCGTTACGGTGTTTTTGTGTAATTAAGACTAGGGCTTTGAACAAAACACTTTCAACTTTGTGAGGTTCGCACCAGTCCTTTTGCCTCACACGCATTTGTTTATTTTTCACGTGCGCGTAGTTCGCTCATTGCTTTCATCCCGCTTTTCGCCGCTTCTTTCTGGGGTTTCTTCCTCTTTTTTTGTGACAATAGACGGTCCATTCTAATCGGGTTTCAAAGTAAATAGCAGCGCAGGGGCGAGAGCAATGTAAATTGTGCTTGTCAGAGCCCTCAGTCGTAGGTAGCGAGGAGCGAGGACTCTAACCAATGGAGTGAAGGAGGCTTGGCTAACCTTAGCCTCCCATTTTCTCTCTCCCCCCTCGATGCAGGGTTCTGACCAGTCAGTCGCCTTTGATTATCAGTTGCCAGCAGCCCCTCTCTTGGCTCCTTGACACGAGCACCATATAAGGCGCAGCGATCTCCATAGAAACGTGTCAGTTTCAATAGTAGTGTCAAAGTTCACTATATACAGACATTCGCGCAGATCTCCGTTTCGGAAACATTGCTCCGCTGGTACTCCCGTTAAAACGCATTCTTTTTTGGGTCTCTGCTTCTTACATATTCCATTAGTTGCTTTTTTTCTTTTCTTTTCCTACTGGAGAGGTGAAACTACATAGCCAGTCGGGCGACGTTGCTTTTTTTCGCTGGACCAGATGAGCTTTATTCATGAACATAGATAGAGAAATCCGTTCTTCAGTGTTTCCTCCTCTCCAACCGCGAAGACGGAGAGAGGAGCAAGGAAGAAAAAAGAGGGGAATTTATTTTGCACAGCACTTTGGATCTGCGGTCCACCAGAAAGCTATTATTTTTGCTTCAACGTGAAGATTTTGTTTTTTACTGCGGTATTTTTTAAGAAAACTGTTTTTTTTATATTACGTCTGGGATCGCTTTCTTCATTCACGATTGGGTTCCCGAATGGCTGACTGCAATTTACCTTGGAACTGGCCTCCCGACAACTGCATATCCTGATCGGGTGCCTTTCTATCGACTCCGGTATTTTGAATGTATTGACCATTTTCTGCTTCTACTTTTTTCCCTATGAGATTGAGTGCTCCGATTTGAATTCGCGCTGCCGTTCGTCCAAGACTTCCCTTTT
